# Knowledge, attitudes, and practices of primary health care physicians regarding the pre-travel counselling of patients with type 2 diabetes in Riyadh, Saudi Arabia

**DOI:** 10.1186/s12875-020-01273-z

**Published:** 2020-09-24

**Authors:** Rabia Khalid Alduraibi, Turky H. Almigbal, Abdullah A. Alrasheed, Mohammed Ali Batais

**Affiliations:** 1 King Fahad Specialist Hospital, Buraydah, Saudi Arabia; 2 Department of Family and Community Medicine, King Khalid University Hospital, Box 2925, Riyadh, PO 11461 Saudi Arabia; 3 Department of Family and Community Medicine, King Saud University, Riyadh, Saudi Arabia

**Keywords:** Knowledge, Attitude, Diabetes, Pre-travel counselling, Saudi traveller

## Abstract

**Background:**

Travel has become an integral part of Saudi life. People with diabetes face many challenges while travelling that can have detrimental effects on glycaemic control. However, no previous studies have investigated pre-travel counselling in Saudi Arabia. This study aims to assess the knowledge, attitudes and practices of primary health care (PHC) physicians regarding pre-travel counselling for patients with type 2 diabetes.

**Methods:**

This cross-sectional study was conducted in PHC centres under the Ministry of Health in Riyadh, Saudi Arabia, during the period 2018–2019. A cluster multistage random sampling technique was used to recruit physicians. The data were collected through a self-administered questionnaire.

**Results:**

Three hundred and eighty-five primary health care physicians were recruited. This study showed that more than half (57.9%) of PHC physicians had poor knowledge scores. Additionally, the following characteristics were significantly associated with poor knowledge: being younger in age, being male, being Saudi, being a general practitioner, and having limited (0–5 years) experience. A total of 183 (47.5%) subjects showed disagreement attitudes towards the importance of pre-travel counselling among patients with diabetes. Furthermore, these disagreement attitudes were significantly associated with being older and having more years of experience. The majority (62.6%) of the physicians had poor practice scores. Poor practices were detected among physicians who were younger, male, and Saudi and who had a general practitioner specialty and degree.

**Conclusions:**

It could be concluded that a gap was detected in the knowledge and practices of primary health care physicians regarding pre-travel counselling for people with type 2 diabetes. Therefore, it is necessary to create easily accessible travel medicine education programmes for Saudi PHC providers to improve the management of travellers with diabetes.

## Background

Over the last few decades, the number of travellers on international flights has been rising, and the prevalence of travel-acquired illness is likely to rise in proportion to the predicted increase in international travel [1].

Travel has become an integral part of Saudi life. Whether for pleasure or work, millions of Saudis travel outside their country every year. Travelling abroad, especially to a developing country, involves a health risk, and according to international surveys, over half of travellers could face a health problem during or after their trip. Travel places an individual in unfamiliar environments, which can be especially challenging for those with chronic diseases such as diabetes [2, 3].

A number of studies have shown that the choice of travel destination by patients with diabetes may be affected by their use of insulin, and some of those patients avoid international travel altogether because of their disease [4, 5].

While travelling, people with diabetes face many challenges that can affect their glycaemic control, including changes in their routines regarding diet and physical activity, and difficulties in adjusting insulin dose and timing after crossing multiple times zones, as well as the risk of infectious diseases, especially when travelling to developing countries [5, 6].

Many travellers with diabetes are not aware of this health risk. Therefore, pre-travel advice provided by primary health care (PHC) physicians is important in maintaining travellers’ health during their journey. Travel health advice is a critical step in a pre-travel consultation. Unfortunately, the uptake of pre-travel health advice remains low, even though most travel-related health problems can be prevented with high-quality medical consultations that include information on lifestyle and dietary rules or the prescription of appropriate medications [2, 3, 6, 7].

Few published health studies have examined physicians’ knowledge and practices regarding travel and diabetes. To the best of our knowledge, no study has focused on travel health practices in Saudi Arabia or even in the Middle East at large. Therefore, our objective is to assess the knowledge and attitudes of PHC physicians regarding pre-travel counselling for patients with type 2 diabetes and to examine the association between the physicians’ demographic characteristics and their knowledge and attitudes.

## Methods

### Study setting

A cross-sectional study was conducted using a self-administered questionnaire among primary health care (PHC) physicians working at primary health care centres (PHCCs) under the Ministry of Health (MOH) in Riyadh, Saudi Arabia, during the period from September 2018 to March 2019.

According to the database of the General Directorate of Health Affairs in Riyadh, approximately 656 physicians work in 135 PHCCs of the MOH in the city of Riyadh. All Primary care physicians (PCPs) working in PHCCs under the MOH in Riyadh were eligible for inclusion. All specialists who were not in direct contact with patients with diabetes, physicians who were on an extended leave of duty, and physicians who declined to participate were excluded.

A cluster multistage random sampling technique was employed. For the purpose of the study, PHCCs were clustered according to the city’s geographic divisions into five regions (middle, southern, northern, eastern, and western), with 18–34 PHCCs in each region. Of these PHCCs, 10 PHCCs in each region were randomly chosen. Therefore, 50 PHCCs were included in the study.

### Sample size

The sample size was calculated using a standard sample size equation “*n* = z^2^p(1-p)/e^2^” and an assumed proportion of 50% (proportion of medical physicians who had correct knowledge regarding pre-travel counselling for patients with diabetes). Using a 95% confidence interval and a 5% margin of error, the sample size was estimated to be 385 and was adjusted to 410 to compensate for the non-response rate.

### Participants and survey instrument

All physicians present at the time of data collection in the selected PHCCs were included; hard copies of the questionnaires were delivered to the available physicians. Without seeing it first, the physicians were asked to complete the anonymous self-administered survey in English in order to assess their basic background knowledge.

The self-administered questionnaire was developed by the principal investigator based on the study objectives and after a literature review of similar studies [8–10]. A panel of two diabetologists and one family physician, all of whom provide clinical care for patients with diabetes and are familiar with diabetes guidelines and the survey’s development, assessed the questionnaire for appropriateness, accuracy, and relevance and were asked to critique the questionnaire’s content. To ensure the face validity of the questionnaire, it was presented to a sample of 20 participants in a pilot study and then finalized. The results of the piloted questionnaires were not included in the analysis.

The questionnaire is divided into four sections with a total of 24 questions. The first section pertains to demographic characteristics, including age, gender, level of education, and nationality.

The second section assesses the physicians’ knowledge regarding diabetes and travel. The response choices for knowledge items include “yes”, “no” and “do not know”. Correct answers were scored as 1, while incorrect answers and “do not know” were scored as 0. The total knowledge score ranges from 0 to 10 (10 items).

After calculating the knowledge score for each participant, we extracted descriptive statistics and found that the score ranged between 2 as a minimum score and 10 as a maximum score, after that the range was calculated, which is the difference between the highest score and the lowest score (10–2 = 8), then knowledge levels were classified as follows: from (2 to 5) consider Poor knowledge and from (6 to 8) consider medium level and from (9 to 10) consider high level of knowledge.

The third section assesses the attitudes of physicians towards pre-travel counselling for patients with diabetes. Five-point Likert scale items were used; strongly agree responses were scored as 5, agree as 4, uncertain as 3, disagree as 2, and strongly disagree as 1. The total attitudes score ranges from 6 to 30 (6 items), with higher scores indicating a higher degree of agreement. The fourth section assesses the practices of physicians towards pre-travel counselling.

Physicians at or above the mean score were considered to present good knowledge, high attitudes, or optimal practices, while those under the mean score were categorized as showing poor knowledge, low attitudes, or poor practices.

### Data management and analysis plan

Data were coded and entered using the Statistical Package for Social Sciences Version 22 (IBM Corporation, Armonk, NY, USA). Categorical data were presented as numbers and percentages and were analysed using the chi-square test. Knowledge, attitude, and practice (KAP) scores were calculated; scores ≥80% were considered good, scores of 60- < 80% were considered moderate, and scores < 60% were considered poor. Continuous data were tested for normality by using the Shapiro-Wilk test. Data that were not normally distributed were expressed as medians and interquartile ranges (expressed as 25th–75th percentiles). Continuous, non-normally distributed independent data were analysed using the Mann-Whitney U test, whereas continuous, non-normally distributed paired data were analysed by using Friedman’s two-way analysis of variance by ranks. *P* ≤ 0.05 was considered statistically significant.

### Ethical considerations

Approval for the study was obtained from the Institutional Review Board, College of Medicine, King Saud University (no. E-18-0488), Riyadh, Saudi Arabia. Official approval letters were obtained from the Directorate of Health Affairs in Riyadh. Each participant received the questionnaire and was informed about the objective of the present study. All participants provided informed written consent before the completion of the questionnaires. The Institutional Review Board has agreed that completing the questionnaire will imply consent.

## Results

### Sample characteristics

Three hundred and eighty-five of 410 primary health care physicians completed the questionnaires (response rate of 94%).

Table 1 shows participants’ socio-demographic characteristics. 207 (53.8%) of PCPs were aged ≤35 years, 178 (46.2%) were aged > 35 years. As the ages had extreme values we chose the age value 35 as a cutoff point as it was the median value. More than half of the participants (56.4%) were females, and 47.3% of the participants were Saudi physicians. The participants were either general practitioners (54.5%) or family medicine physicians (45.5%). Most of the respondents were general practitioners and residents (54.5 and 27.8%, respectively), whereas registrars and consultants were less common (16.1 and 1.6%, respectively). Regarding work experience, the majority (39.7%) had 0–5 years of experience, and fewer participants had 6–10 years (19.5%), 11–15 years (20%), or > 15 years (20.8%) of experience.

**Table 1:** Socio-demographic characteristics

Categorical variables		N	%
Age	≤35	207	53.8%
	> 35	178	46.2%
Sex	Female	217	56.4%
	Male	168	43.6%
Nationality	Non-Saudi	203	52.7%
	Saudi	182	47.3%
Specialty	Family Medicine	175	45.5%
	General Practitioner	210	54.5%
Degree of Education	General Practitioner	210	54.5%
	Resident	107	27.8%
	Register	62	16.1%
	Consultant	6	1.6%
Years of Practice	0–5	153	39.7%
	6–10	75	19.5%
	11–15	77	20.0%
	> 15	80	20.8%

### Knowledge of diabetes and travel

The majority (96.4%) of PCPs had adequate knowledge that patients with diabetes should be advised to carry medicines and carbohydrate-rich snacks in easily accessible bags while travelling. In contrast, only two-thirds (60.8%) knew that insulin should not be stored in checked luggage. Moreover, more than half of physicians (56.9%) did not know that travelling across more than five time zones requires adjustment of insulin dose and frequency, while only approximately one third (32.2%) knew that travelling across more than five time zones does not require adjustment of oral anti-hypoglycaemic dose. Few PCPs knew that patients with diabetes travelling to the east region may need to increase their insulin dose, while those travelling to the west region may need to decrease their insulin dose (6.8 and 6.2%, respectively). A total of 212 (55.1%) physicians were aware of the effect of hot or cold climates on insulin and blood glucose monitoring while travelling. The majority (96.6%) recognized the importance of pre-travel vaccination, and all PCPs realized that patients with diabetes need to carry their diabetes ID while travelling abroad. Participants’ knowledge was deficient regarding the need to avoid injecting insulin while a plane is taking off (26.2%) (Table 2).

**Table 2:** Practitioners’ knowledge of pre-travel counselling (*N* = 385)

Question	N	%
1- Correctly answered that patients with diabetes should be advised to carry medicines and carbohydrate-rich snacks in easily accessible bags while travelling	371	96.4%
2- Correctly answered that insulin cannot be stored in checked luggage.	234	60.8%
3- Correctly answered that, during air travel, patients with diabetes are advised to not inject insulin at take-off.	101	26.2%
4- Correctly answered that travelling across more than five time zones requires adjustment of insulin dose and frequency.	166	43.1%
5- Correctly answered that travelling across more than five time zones does not require adjustment of oral anti-hypoglycaemic dose.	124	32.2%
6- Correctly answered that patients with diabetes who are travelling to the east region may need to increase their insulin dose	26	6.8%
7- Correctly answered that patients with diabetes who are travelling to the west region may need to decrease their insulin dose.	24	6.2%
8- Correctly answered that extremes of hot or cold climates can affect insulin and blood glucose monitoring in patients with diabetes while travelling.	212	55.1%
9- Correctly answered that pre-travel vaccination is important for patients with diabetes.	372	96.6%
10- Correctly answered that patients with diabetes need to carry ID that says that they have diabetes while travelling abroad.	385	100.0%

The total knowledge score ranged from 2 to 10, with a median of 5 (IQR = 4–6). More than half (57.9%) of the participants had poor scores, and 3.6% had good scores. Table 3 demonstrates a statistically significant association between the total knowledge score and age, sex, nationality, level of education and years of experience. Practitioners’ nationality had strongly significant association with knowledge regarding diabetes and travel; since 130 out of 180 Non-Saudi which represents (72.22%) of Non-Saudi physicians had poor knowledge regarding diabetes and travel verses only 93 out of 203 Saudi physicians which represents (45.81%) of Saudi physicians. Significant associations were found (*P* < .05) between poor knowledge and the following participant characteristics: being younger than 35 years old, being male, being Saudi, being a general practitioner and having limited (0–5 years) experience.

**Table 3:** Association between knowledge score and socio-demographic data

		Knowledge Score								
		Good *N* = 14 (3.6%)		Moderate *N* = 148 (38.4%)		Poor *N* = 223 (57.9%)		Total *N* = 385		Chi-Square Test
		N	%	N	%	N	%	N	%	*P* value
Age	≤35	2	14.3%	61	41.2%	144	64.6%	207	53.8%	<.001*
	> 35	12	85.7%	87	58.8%	79	35.4%	178	46.2%	
Sex	Female	8	57.1%	99	66.9%	110	49.3%	217	56.4%	.004*
	Male	6	42.9%	49	33.1%	113	50.7%	168	43.6%	
Nationality	Non-Saudi	12	85.7%	98	66.2%	93	41.7%	203	52.7%	<.001*
	Saudi	2	14.3%	50	33.8%	130	58.3%	182	47.3%	
Specialty	Family Medicine	4	28.6%	59	39.9%	112	50.2%	175	45.5%	.063
	General Practitioner	10	71.4%	89	60.1%	111	49.8%	210	54.5%	
Education Degree	General Practitioner	10	71.4%	89	60.1%	111	49.8%	210	54.5%	<.001*
	Resident	2	14.3%	23	15.5%	82	36.8%	107	27.8%	
	Register	2	14.3%	34	23.0%	26	11.7%	62	16.1%	
	Consultant	0	0.0%	2	1.4%	4	1.8%	6	1.6%	
Years of Practice	0–5	2	14.3%	42	28.4%	109	48.9%	153	39.7%	<.001*
	6–10	0	0.0%	25	16.9%	50	22.4%	75	19.5%	
	11–15	4	28.6%	36	24.3%	37	16.6%	77	20.0%	
	> 15	8	57.1%	45	30.4%	27	12.1%	80	20.8%	

*significant at *p* < .05

### Physicians’ attitudes towards pre-travel counselling and diabetes

The participants’ attitudes towards pre-travel counselling are illustrated in Table 4. Most of them strongly agreed with the following statements: pre-travel counselling for patients with diabetes is important; the availability of an Arabic resource to increase patients’ awareness of health practices before, during and after the trip is needed; and I would advise patients to visit such a resource (90.4, 83.4, and 83.9%, respectively). Moreover, the majority (77.7%) of PCPs strongly agreed that seeking medical advice before travelling would decrease patients’ chances of getting sick during their trip. Furthermore, more than half (57.1%) strongly agreed that Saudi Arabia lacks travel medicine practices, and two-thirds (60%) strongly agreed that our society lacks knowledge of the importance of travel medicine.

**Table 4:** Practitioners’ attitudes towards pre-travel counselling (*N* = 385)

Question		N	%
1- Pre-travel counselling for patients with diabetes is important.	Agree	37	9.6%
	Strongly agree	348	90.4%
2- The availability of an Arabic resource to increase patients’ awareness of health practices before, during and after the trip is needed.	Agree	64	16.6%
	Strongly agree	321	83.4%
3- If there is a trusted Arabic resource to increase patients’ awareness of health practices before, during and after the trip, I will advise my patient to visit it.	Agree	62	16.1%
	Strongly agree	323	83.9%
4- Patients who seek medical advice before travelling will have lower chances of getting sick during their trip.	Agree	86	22.3%
	Strongly agree	299	77.7%
5- In Saudi Arabia, we lack the practice of travel medicine.	Agree	159	41.3%
	Strongly agree	220	57.1%
6- Our society lacks knowledge of the importance of travel medicine.	Agree	146	37.9%
	Strongly agree	231	60.0%

The total attitudes score ranged from 0 to 6 with a median of 5 (IQR =4–6). More than half (52.5%) of the participants had strong agreement attitudes, while 183 (47.5%) showed disagreement attitudes towards the importance of pre-travel counselling among patients with diabetes. Table 5 shows that significantly higher percentages of physicians with disagreement attitudes were older than 35 years, whereas most participants who showed agreement were younger (*p* = .003). In addition, years of experience were significantly higher among physicians with disagreement attitudes (*p* = .006).

**Table 5:** Association between attitudes score and socio-demographic data

		Attitudes score						
		Disagree *N* = 183 (47.5%)		Agree *N* = 202 (52.5%)		Total *N* = 385		
		N	%	N	%	N	%	*P* value
Age	≤35	84	45.9%	123	60.9%	207	53.8%	.003*
	> 35	99	54.1%	79	39.1%	178	46.2%	
Sex	Female	103	56.3%	114	56.4%	217	56.4%	.98
	Male	80	43.7%	88	43.6%	168	43.6%	
Nationality	Non-Saudi	103	56.3%	100	49.5%	203	52.7%	.183
	Saudi	80	43.7%	102	50.5%	182	47.3%	
Specialty	Family Medicine	83	45.4%	92	45.5%	175	45.5%	.97
	General Practitioner	100	54.6%	110	54.5%	210	54.5%	
Education Degree	General Practitioner	100	54.6%	110	54.5%	210	54.5%	.12
	Resident	46	25.1%	61	30.2%	107	27.8%	
	Register	36	19.7%	26	12.9%	62	16.1%	
	Consultant	1	0.5%	5	2.5%	6	1.6%	
Years of Practice	0–5	61	33.3%	92	45.5%	153	39.7%	.006*
	6–10	33	18.0%	42	20.8%	75	19.5%	
	11–15	38	20.8%	39	19.3%	77	20.0%	
	> 15	51	27.9%	29	14.4%	80	20.8%	

*significant at *p* < .05

### Management practices

The relationship between physicians’ practices towards pre-travel counselling and their degree of education is illustrated in Table 6. The majority (46.5%) of the participants reported that 20–40 patients with diabetes visit the clinic weekly for any reason. However, approximately 53% reported that only 1–10 patients per month ask for advice before travelling; this number was significantly increased among general practitioners (*p* < .001). Two hundred and thirty (59.7%) participants reported that pre-travel counselling would take 5–15 min. A significantly higher percentage of these physicians were consultants (*p* < .001). Additionally, the majority (84.2%) of respondents reported that they would advise and counsel patients with diabetes regarding the importance of recommended vaccines before they travelled. More than half (59.0%) were aware of travel safety recommendations for patients with diabetes. A significantly higher percentage of these physicians were general practitioners (*p* < .001). Approximately two-thirds (67.8%) reported that they did not feel confident about how to adjust insulin doses for patients travelling across several time zones. A significantly higher percentage of these physicians were general practitioners (*p* < 001). A total of 219 (56.9%) participants stated that patients mostly asked about diabetes IDs, vaccinations, prescriptions, and medication adjustments.

**Table 6:** Practitioners’ practices towards pre-travel counselling and their relation to their degree of education

	Education degree					
	GP	Resident	Register	Consultant	Total	
	N (%)	N (%)	N (%)	N (%)	N (%)	*P* value
1- Estimated number of patients with diabetes that visit clinic per week for any reason 20–40	100 (47.6%)	47 (43.9)	28 (45.2%)	4 (66.7%)	179 (46.5%)	.77
2- Estimated number of patients with diabetes that ask for advice before his/her trip per month 1–10	128 (61.0%)	34 (31.8%)	38 (61.3%)	5 (83.3%)	205 (53.2%)	<.001*
3- Counseled a patient with diabetes before traveling, it take about 5–15 min	145 (69.0%)	46 (43.0%)	34 (54.8%)	5 (83.3%)	230 (59.7%)	<.001*
4- I advise and counsel patient with diabetes regarding the importance of vaccines before travel	174 (82.9%)	86 (80.4%)	58 (93.5%)	6 (100%)	324 (84.2%)	.08
5- I face patients with diabetes who are trying to avoid travel because of their illness	50 (23.8%)	22 (20.6%)	14 (22.6%)	2 (33.3%)	88 (22.9%)	.85
6- I am aware of travel safety recommendations for patients with diabetes	143 (68.1%)	34 (31.8%)	46 (74.2%)	4 (66.7%)	227 (59.0%)	<.001*
7- I don’t feel confident about how to adjust insulin dose for patients who travel across several time zones	123 (58.6%)	97 (90.7%)	38 (61.3%)	3 (50.0%)	261 (67.8%)	<.001*

*significant at *p* < .05

The mean ranks of knowledge scores were significantly higher among physicians who were older than 35 years of age, were female, were non-Saudi, were general practitioners, and were at the registrar rank (*p* < .05). Similarly, the practice score was significantly higher among physicians who were older than 35 years, were female, were non-Saudi, were general practitioners, and were consultants. However, the mean rank of the attitudes score was significantly higher among physicians aged 35 or younger (*p* = .038), as demonstrated in Table 7.

**Table 7:** Comparison of demographic characteristics and KAP scores

		Knowledge score			Attitudes score		Practice score	
		Mean rank		*P* value	Mean rank	*P* value	Mean rank	*P* value
Age	<=35	166.56		<.001*	203.45	.038*	160.61	<.001*
	> 35	223.75			180.84		230.67	
Sex	Female	208.59		.001*		.074	215.51	<.001*
	Male	172.86					163.92	
Nationality	Non-Saudi	220.55		<.001*		.67	224.40	<.001*
	Saudi	162.27					157.98	
Specialty	Family Medicine	175.16		.003*		.71	166.03	<.001*
	General Practitioner	207.87					215.48	
Degree of Education	General Practitioner		207.87	<.001*		.28	215.48	<.001*
	Resident		147.45				134.77	
	Register		223.16				212.34	
	Consultant		173.33				244.50	

*significant at *p* < .05

The comparison of the three scores among the studied participants revealed a significantly higher knowledge score, followed by the attitudes score and the practice score (mean ranks were 2.54, 2.19 and 1.27, respectively), as shown in Table 8.

**Table 8:** Score summary of knowledge, attitudes, and practices among primary health care physicians towards pre-travel counselling

Scores		N	%	Minimum- Maximum	Median	IQR	Mean Ranks
Knowledge	Good	14	3.6%	2–10	5	4–6	2.54
	Moderate	148	38.4%				
	Poor	223	57.9%				
Attitude	Disagree	183	47.5%	0–6	5	4–6	2.19
	Agree	202	52.5%				
Practice	Good	144	37.4%	1–4	3	2–4	1.27
	Poor	241	62.6%				

Table 9 illustrates a significant association between knowledge and practice scores (*p* < .001), while the attitudes score did not show a significant relationship (*p* > .05).

**Table 9:** Association between knowledge and subjects’ attitudes and practices towards pre-travel counselling

		Knowledge score						
		≤5		> 5		Total		
		N	%	N	%	N	%	*P* value
Attitudes	Disagree	102	55.7%	81	44.3%	183	100.0%	.41
	Agree	121	59.9%	81	40.1%	202	100.0%	
Practices	Good	56	38.9%	88	61.1%	144	100.0%	<.001*
	Poor	167	69.3%	74	30.7%	241	100.0%	

*significant at *p* < .05

## Discussion

To our knowledge, this study was the first to assess the knowledge, attitudes and practices of general health care providers regarding the pre-travel counselling of patients with diabetes in Saudi Arabia and the Middle East at large. It has demonstrated essential findings. First, this study showed that more than half (57.9%) of the participants had poor knowledge scores. Second, more than half (52.5%) of participants had agreement attitudes, while 183 (47.5%) showed disagreement attitudes towards the importance of pre-travel counselling for patients with diabetes. Third, the majority (62.6%) of the participants had low practice scores.

Travel health advice for patients with diabetes can be complex. Understanding the demographic features and travel-associated risk factors is important. While most participants identified the importance of pre-travel counselling of patients with diabetes, we found some important knowledge gaps. Participants were generally able to clinically advise patients with diabetes that they should to carry medicines and carbohydrate-rich snacks in easily accessible bags while travelling.

While the majority of participants in our study did not know that travelling across more than five time zones and in hot or cold climates affected insulin dose and frequency. Few participants knew that patients with diabetes travelling to the east region may need to increase their insulin dose, while those travelling to the west region may need to decrease their insulin dose. Similar to our findings, in a study by Gill and Redmond [6], investigators sent questionnaires to 160 physicians and asked how they were advising patients on insulin adjustments while traveling. They received questionnaires back from 60 (37%) physicians. They study showed that advice was being given by the majority of these clinics with only 3% not giving any specific advice. Unfortunately, 21% of the advice provided was judged to be either unhelpful or potentially harmful.

Likewise, Piotte et al. [1] assessed the level of specific knowledge among primary care providers in eastern France regarding health advice, vaccinations and malaria prophylaxis. They concluded that the participants’ high level of knowledge in travel medicine was mostly linked to their motivation to practice in this specialized discipline. This finding should be considered with respect to the provision of education programmes in our society.

Another study by Al-Hajri et al. [11] surveyed 76 PHC physicians in Qatar. The questionnaire included items assessing socio-demographic characteristics and knowledge and practices related to travel medicine before and after an educational symposium [11]. They detected significantly increased knowledge on the post-symposium questionnaire for most questions. This reflects the fact that the availability of extensive educational programs for healthcare providers will increase their awareness and knowledge.

Regarding the attitudes of participants towards pre-travel counselling, our results showed that more than half of participants strongly agreed that Saudi Arabia lacks the practice of travel medicine, and two-thirds strongly agreed that our society lacks knowledge of the importance of travel medicine. As travel becomes more frequent in Saudi, pre-travel counselling and risk assessment are needed to understand travel-related risks and to better enable preparation for such activity. Therefore, our findings could promote the implementation of training programmes on travel medicine to cover the deficiencies detected and to provide sufficient information during pre-travel counselling.

With regard to the practice of pre-travel counselling, our results showed that approximately two-thirds of the participants, especially general practitioners, reported that they did not feel confident about how to adjust insulin doses for patients travelling across several time zones. This may be the reason that most provider characteristics were not associated with the knowledge of guideline recommendations for travel health. Additionally, there is a lack of health travel programmes.

Similar to our findings, Kogelman et al. [12] compared the knowledge, attitudes and practices of US primary care providers and US travel medicine specialists. They demonstrated knowledge and practice deficits among practitioners offering travel medicine advice. Furthermore, they revealed that familiarity with travel-specific vaccines and knowledge scores based on brief pre-travel scenarios were higher among travel medicine specialists.

The provision of comprehensive pre-travel health advice is essential to reduce the incidence of travel-related morbidity. Studies from other countries found that primary care physicians are active in travel medicine [13]. In our study more than half (84.2%) of the PHCP counselling patients with diabetes regarding the importance of vaccines before travel. This reflects the fact that the available vaccination schedules are better known.

In contrast to the study was done in Qatar, which reported that more than 50% did not offer travel health counselling for traveller [11].

A comparison of the knowledge, attitudes and practices scores among the studied participants revealed a significantly higher knowledge score, followed by the attitudes score and the practice score (mean ranks were 2.54, 2.19 and 1.27, respectively). Additionally, a significant positive correlation was detected between knowledge and practice scores. Improving the level of knowledge of PHC physicians directly affects their practices. Furthermore, the structure of pre-travel consultations should address the travellers’ wishes, expectations, difficulties, experiences, and previous knowledge. Physicians should ask the traveller whether he or she understood the advice given. Finally, a booklet with additional advice and a website where patients can find health advice on their destination should be provided [14].

A limitation of this study is the use of a survey tool that has not undergone prior reliability and validity testing. In addition, the result of this study cannot be generalised to other populations in the country because KAP might be greatly influenced by socio-demographic factors of the population. More studies on the travel of Saudi’s population with diabetes need to be performed, especially with the increasing affluence and diverse travel habits in the region.

## Conclusions

The study has found a gap in the knowledge and practices of primary health care physicians regarding pre-travel counselling for patients with type 2 diabetes. In addition, these deficiencies were more significant among physicians who were younger, male, and Saudi and who had a general practitioner specialty; in contrast, disagreement attitudes were significantly associated with being older and having more years of experience. Therefore, easily accessible travel medicine education programmes for Saudi PHC providers are needed to improve the management of travellers with diabetes.

## Data Availability

The data generated or analyzed during this study were included in this published article.

## Abbreviations

PHC: Primary health care
PHCCs: Primary health care centres
MOH: Ministry of health
PCPs: Primary care physicians
KAP: Knowledge, attitude, and practice
